# Uptake of routine vitamin A supplementation for children in Humbo district, southern Ethiopia: community-based cross-sectional study

**DOI:** 10.1186/s12889-020-09617-1

**Published:** 2020-10-02

**Authors:** Getnet Kassa, Addisalem Mesfin, Samson Gebremedhin

**Affiliations:** 1 Southern Nations Nationalities and People’s Region, Health Bureau, Hawassa, Ethiopia; 2 School of Nutrition, Food Science and Technology, Hawassa University, Hawassa, Ethiopia; 3 School of Public Health, Addis Ababa University, Addis Ababa, Ethiopia

**Keywords:** Vitamin A supplementation, Vitamin A, Uptake of health service

## Abstract

**Background:**

In low- and middle-income countries routine vitamin A supplementation (VAS) is a key strategy for reducing vitamin A deficiency and mortality and morbidity of preschool children. However, in Ethiopia, there is paucity of evidence regarding the level and determinants of the uptake of the supplement. This study was designed to assess the coverage and predictors of VAS among preschool children in Humbo district, Southern Ethiopia.

**Methods:**

A cross-sectional study was conducted in April 2016. A total of 840 mothers/caregivers having children 6–59 months of age were selected using multistage cluster sampling technique from six rural villages implementing routine VAS program. Data were collected using interviewer administered questionnaire. Possible predictors considered in the study include distance from the nearby health facility, household socio-economic status, type of the household (model vs non-model), maternal access to health education on VAS, and knowledge on vitamin A and VAS. Multivariable logistic regression analysis was performed to identify predictors of uptake of VAS. The outputs are presented using adjusted odds ratio (AOR) with the respective 95% confidence interval (CI).

**Results:**

The coverage of VAS was 75.0% (95% CI: 72.1–77.9). Better knowledge of mothers about the importance of the supplement (AOR: 1.49, 1.02–2.17), obtaining VAS related information from frontline community health workers (AOR: 1.51, 1.34–2.72) than health professionals and being from households in the “rich” wealth tertile (AOR: 1.80, 95% CI: 1.07–3.03) were positively associated with uptake VAS.

**Conclusion:**

The VAS coverage of the area was approaching the expected national target of 80%. However, the uptake can be enhanced though awareness creation and improving socio-economic status of the community.

## Background

Vitamin A plays an indispensable role in diverse physiological functions including vision, immunity and growth [1]. Vitamin A deficiency (VAD) – serum retinol level < 0.70 μmol/l – is a major health problem affecting more than one-third (equivalent to 190 million) of the global preschool children. About five million preschool children are also affected by night blindness. Based on the extent of the problem, VAD has moderate or severe public health significance in more than 120 countries. Africa and South-East Asia countries take the highest burden of the deficiency [2].

VAD seriously limits the wellbeing and survival of children [1, 2]. The deficiency is the single most important cause of preventable blindness in the developing world and increases risk of child mortality and morbidity secondary to diarrhea and measles [1, 3]. Systematic review of vitamin A supplementation (VAS) trials concluded that vitamin A reduces all-cause child mortality and diarrhea-related mortality by nearly a quarter [3, 4]. It also substantially cuts the incidence of diarrhea, measles and xerophthalmia [3].

In Ethiopia, despite the decline witnessed in the last three decades, child mortality remains high. According to the recent Demographic and Health Survey (DHS) 2016, out of 1000 live-births, 48 and 67 die before celebrating their first and fifth birthdays, respectively [5]. A national survey conducted in 2007 concluded that 38% of children 6 to 71 months had low serum vitamin A levels and 1.7 had Bitot’s spots [6]. Another large-scale study conducted in 238 Community Based Nutrition program implementing districts of the country estimated that VAD caused 80,000 deaths in a year and affected 61% of children under the age of 5 years [7, 8].

VAS is considered as a quick and cost-effective strategy for improve VA status in settings where VAD is a public health problem [9]. The World Health Organization (WHO) recommends for the routine administration of VAS to children 6–59 months twice a year [9]. VAS coverage of 80% or more is necessary to achieve the child mortality reduction of the intervention [10]. However, the global coverage remains unsatisfactory. A recent estimate based on several DHS surveys indicated that in Sub-Saharan Africa nearly half of pre-school children did not get the supplement [11].

In Ethiopia routine VAS program has been in place for about two decades and the National Nutrition Program (NNP) promotes the distribution of the supplement to children [8]. In the last two decades, the supplementation had been actively implemented at grassroots level via the routine Health Extension Program (HEP) and other campaign-based approaches – Enhanced Outreach Strategy (EOS) and Community Health Day (CHD) modalities. However, the coverage remains unsatisfactory. According to the Ethiopian DHS 2016, 45% of the eligible children received the supplement in the preceding 6 months of the survey [5]. In 2011, the national coverage was even higher (56%) [12]. Since 2010, the delivery of vitamin A supplements in Ethiopia had gradually shifted from campaign-based approach to routine delivery via the HEP [13].

So far limited evidence exists regarding the factors that affect the utilization of VAS in Ethiopia. Accordingly, this study was conducted to assess the coverage of and factors associated with uptake of routine VAS in Humbo district, Southern Ethiopia. Especially, the study was relevant as it was carried out after transition of VAS distribution from campaign-based approach to routine delivery via the HEP was completed in the district.

## Methods

### Study setting

This study was conducted in April 2016 in Humbo district, Southern Ethiopia. The district has 39 rural and 5 urban *kebeles* (the smallest administrative unit in Ethiopia having approximately 1000 households). Humbo has a total population of 157,073 of which children 6–59 months of age contribute to 11% of the population. In the district there are 6 health centres and 39 health posts. About 78 health extension workers (HEWs) are deployed in the health posts and are providing community-based service including distribution of VAS. HEWs are cadres of trained female community-based health workers who received at least 1 year of training after completing secondary education. In each kebele, on average 2 HEWs are deployed. In every kebele, a network of volunteers, called the Health Development Army (HDA) members support HEWs through community mobilization.

In Ethiopia VAS is distributed through three strategies: Enhanced Outreach Strategy (EOS), Community Health Days (CHDs) and the routine Health Extension Program (HEP). The EOS is a makeshift strategy involving distribution of the supplement via centrally organized semi-annual or quarterly campaigns along with deworming and screening for malnutrition. The CHD is a similar modality as that of the EOS except that campaigns are organized at *kebele* level. The CHD is considered as a transitional stage to the ultimate integration of VAS into the HEP. In the third strategy, VAS is considered as the integral component of the HEP and the supplement is provided by HEWs through a mixture of static service, locally organized campaigns and home visits. In Humbo district, at the time of the survey VAS was mainly distributed via the third option – distribution through routine HEP.

### Study design

We implemented quantitative cross-sectional survey in April 2016 in Humbo district. The source population for the study was all children 6–59 months of age residing in the rural kebeles of the district; whereas, the study population was similar group of children residing in randomly selected six kebeles of Humbo district.

### Sample size and sampling procedure

A sample size of 840 was calculated using single population proportion formula with the inputs of 95% confidence level, 5% margin of error, 53.1% expected coverage of VAS [12] design effect of 2 and 10% compensation for possible non-response.

The study subjects were identified using multistage cluster sampling approach. Initially, the 39 rural kebeles found in the district were categorized as accessible (30 kebeles) and non-accessible (9 kebeles) based on a cut-off distance of 10 kms from the nearest major all-weather roads. Then, accessible and non-accessible kebeles were separately listed and, four and two kebeles were randomly selected from the two strata, respectively. The total sample size of 840 was allotted to each of the six kebeles proportional to their population size and the sample size distributed per kebele ranged from 116 to 174 children. Ahead of the study, complete listing of eligible children was made and used as a sampling frame. Ultimately the required subjects were selected using systematic random sampling technique.

### Data collection tools

Data were collected in April 2016 using pre-tested and structured questionnaire developed by the investigators specifically for the study. The questionnaire used in the study is provided as supplementary file with this manuscript (Supplementary File 1). The questionnaire was developed in English and translated to the local Wolaitigna language. Socio-demographic and economic related information were collected using questions extracted from the standard DHS questionnaire. The DHS questionnaire is a standard tool designed to collect demographic and health data consistently in multiple low-income countries. We collected the data using experienced and trained enumerators and supervisors. The VAS status of the selected child was determined by showing the mother/caregiver a VAS capsule and asking whether the child had received a similar one in the past 6 months.

### Variables of the study

The dependent variable of the study was the recent (last 6 months) vitamin A supplementation status of the child. The independent variables were selected based on previous literature [5, 11, 12] and include socio-demographic variables (maternal age, maternal and paternal educational status, household wealth index, family size, age and sex of the child), type of the household (whether the household had been considered as model or non-model household by local HEWs), distance from the nearest health facility, whether the respondent received health education about vitamin A supplement in the last 12 months or not, knowledge about at least one benefit of vitamin A supplements to children, knowledge about at least one consequence of VAD and knowledge about dietary sources of vitamin A. According to the HEP of Ethiopia, model households are families that successfully implemented all the packages of the health extension program with the support of the community health workers.

### Data processing and analysis

The data were entered, cleaned and analyzed using SPSS for windows, version 20. Frequencies, percentage, mean and standard deviation were used to summarize the data.

Bivariable logistic regression analysis was performed to assess the association of each predictor with the outcome variable and multivariable logistic model was to control the effect of confounders. Variables with *p*-value less than 0.25 in the bivariable models were considered as candidate variables for the multivariable analysis [14]. In the multivariable analysis, proximate and distal variables were fitted separately in order to avoid over-adjustment bias [15]. The distal variables considered in the analysis were: household wealth index, maternal and paternal educational status, household type (being model or non-model household), sex and birth order of the baby. On the other hand, the proximate variables were: knowledge on vitamin A and VAS, physical access to the nearby health institution, exposure to VAS promotion activities and age of the child. Fitness of the logistic model was checked using Hosmer and Lemeshow statistic. Absence of multicollinearity was checked following standard approaches. The outputs of the analysis are presented using crude (COR) and adjusted odds ratio (AOR) with the 95% confidence interval (CI).

Wealth index was constructed as an indicator of household economic status based on variables related to housing conditions, ownership of household assets, type of drinking water source, size of agricultural land, and number of livestock owned. Principal component analysis (PCA) was performed to generate a summary wealth index score and the score was ultimately categorized into three tertiles: poor, medium and rich.

### Ethical considerations

The study was approved by the ethics committee at Hawassa University, College of Medicine and Health Science. Data were collected after security informed verbal consent from the primary caregivers of the study children. Verbal, rather that written consent was used because significant proportion of the population in the area had no formal education. The same was approved by the ethics committee that reviewed the protocol.

## Results

### Socio-demographic and economic characteristics

A total of 813 mothers/caregivers volunteered to participate in the study making the response rate 96.8%. The mean age (±SD) of children was 32.4 ± 14.0 months and 92.7% were between 12 and 59 months of age. Boys and girls were almost equality represented with the ratio of 0.95.

The mean (±SD) age of the mothers was 28.9 ± 4.8 years and the majority (63.9%) were between 25 to 34 years. Pertaining to marital status, nearly all (97.7%) were married or living together with their partners. Two-third (66.2%) of the respondents and 88.3% of their partners were literates. Regarding occupation, nearly three-fourths considered themselves as farmers. The vast majority (98.0%) of the households owned a private house. However, most of the houses had rudimentary structure. In three-fourths of the households (74.5%) the main source of income was farming. Further, 94.8% of the household possessed one hectare or lager plot of agricultural land and 89.5% owned livestock. The majority (97.7%) of the households had access to improved drinking water sources [16] (Table 1).

**Table 1:** Socio-demographic characteristics of the respondents and the index children in Humbo district SNNP, March 2016

Characteristics	Frequency	Percent
Sex of the child (*n* = 813)		
Male	397	48.8
Female	416	51.2
Age of the child (months) (*n* = 813)		
6–11	59	7.3
12–59	754	92.7
Age of the mother (years) (*n* = 813)		
18–24	228	28.0
25–34	519	63.9
35–49	66	8.1
Marital status (*n* = 813)		
Married/living together	794	97.7
Others	19	2.4
Educational status of the mother (*n* = 813)		
No formal education	275	33.8
Elementary school (grade 1–6)	372	45.8
Junior high school (grade 7–8)	123	15.1
High school (grade 9–12) or above	43	5.3
Educational status of the father (*n* = 813)		
No formal education	95	11.7
Elementary school (grade 1–6)	122	15.0
Junior high school (grade7–8)	220	27.1
High school (grade 9–12) or above	376	46.2
Occupation of the mother (*n* = 813)		
Farmer	606	74.5
Housewife	199	24.5
Employed	8	1.0
Family size (*n* = 813)		
1–5	411	50.6
More than 5	402	49.4
Ownership of agricultural land (*n* = 813)		
< =1 ha	771	94.8
> 1 ha	42	5.2
Ownership of private house (*n* = 797)		
Yes	797	98.0
No	16	2.0
Type of roof of the house (*n* = 797)		
No or rudimentary	617	77.4
Finished roof	180	22.6
Type of wall of the house (*n* = 797)		
Rudimentary wall	797	100.0
Finished wall	0	0.0
Type of floor of the house (*n* = 797)		
Natural or rudimentary	794	99.6
Finished floor	3	0.4
Ownership of livestock (*n* = 813)		
Yes	728	89.5
No	85	10.5
Source of drinking water (*n* = 813)		
Improved	794	97.7
Non-improved	19	2.3

### Knowledge about vitamin A

Nearly all (94.6%) of the mothers/caregivers had received information about vitamin A from at least one source in the preceding 6 months of the survey. About half (46.0%) identified at least one rich dietary source of vitamin A. Among them 87.4, 79.9 and 94.4% reported dairy products, eggs and meat, respectively as rich dietary sources. However, very few recognized dark green leafy vegetables (6.4%) or vegetables orange/yellow inside (6.7%) as vitamin A sources.

The majority (94.7%) of the respondents knew at least one manifestation or consequence of VAD. Night blindness was recognized as a symptom by 93.8% of the respondents. Conversely, smaller proportions were aware that the deficiency can cause permanent loss of vision (5.5%), poor physical growth (1.9%), illness (2.2%) or death (1.6%) in children.

Regarding information about VAS, the vast majority (97.2%) had received information about the supplement in the preceding 6 months. The leading sources of information were HEWs (97.5%), health development army (HDAs) members (87.8%) and health professionals (51.9%). Mass media was considered as a source only by one-fifth (20.4%) of mothers/caregivers.

About 97.5% of the respondents had seen VA capsule at least once before. Three-fourth (75.6%) assumed that VAS has importance to children. The majority (99.3%) were aware that it prevents blindness. Conversely, very few were aware that VAS prevents disease (1.8%) and reduces risk of childhood death (0.7%). None identified the importance of the supplementation for fostering physical growth of children (Table 2).

**Table 2:** Knowledge about vitamin A and vitamin A supplementation in Humbo district, March 2016

Knowledge indicators (*n* = 813)	Frequency	Percentage
Ever heard of vitamin A	769	94.6
Knows food sources of vitamin A	374	46.0
Knows at least one consequence of VAD	770	94.6
Have information about VAS	790	97.2
Respondents observed VA capsule before	770	97.5

### Vitamin a supplementation coverage

As described earlier, the VAS status of the study children was determined by showing the mother/caregiver a VAS capsule and asking whether the child had received the same in the past 6 months. Accordingly, 75.0% (95% CI: 72.1–77.9) of the mothers reported that their children received the capsule in the reference period. The VAS coverages among infants 6–11 months (71.1%) and children 12–59 months (75.3%) were not significantly different. Among the supplemented children, 63.1% received the supplement through the routine HEP and 36.9% through campaigns. 7.2% took the supplement through home-to-home visits.

### Factors associated with uptake of vitamin a supplementation

Based on the bivariable analyses, six proximate variables satisfied the candidacy criteria (*p* < 0.25) for the multivariable model. These were: knowledge on importance of VAS, awareness of consequences of VAD, knowledge on dietary sources of vitamin A, time required to travel to the nearest health facility, and type of health worker who provided information about VAS. Similarly, four distal variables (household wealth index, type of the household (model vs non-model household), educational status of mother and her husband) were included in the multivariable model.

In the multivariable model, it was found that mothers who were aware of the benefit of VAS had 1.5 times increased odds of receiving the supplement. Further, mothers who obtained information on VAS from HEWs and HDAs had 1.5 times increased odds of utilizing the service than those who were contacted by health professionals. Children from families in the “rich” wealth tertile had 1.8 times higher odds of receiving VAS than those from the “poor” households. On the other hand, children with mothers having no formal education had increased odds of receiving a vitamin A capsule (Table 3).

**Table 3:** Factors associated with uptake of VAS among children 6–59 months in Humbo district, March 2016

Independent variables	VAS		COR (95% CI)	AOR (95% CI)
	Yes	No		
Information about VA				
Yes	584	185	2.18 (1.17–4.07)	1.05 (0.28–3.95)
No	26	18	1	1
Information about sources of VA				
Yes	289	85	1.25 (0.90–1.73)	1.10 (0.79–1.45)
No	321	118	1	1
VAS capsule observed before				
Yes	598	195	2.04 (0.82–5.07)	1.12 (0.34–3.68)
No	12	8	1	1
Information about effect of VAD				
Yes	584	186	2.05 (1.09–3.86)	1.94 (0.53–7.13)
No	26	17	1	1
Information about importance of VAS				
Yes	476	139	1.63 (1.04–2.32)*	1.49 (1.02–2.17)*
No	134	64	1	1
Sources of information about VAS				
Health professionals	126	67	1	1
HEWs and HDA	484	136	1.89 (1.33–2.69)*	1.51 (1.34–2.72)*
Time to reach to the nearest health facility				
Less than 30 min	631	182	0.73 (0.50–1.05)	1.45 (0.98–2.14)
More than 30 min	182	631	1	1
Educational status of the respondent				
Illiterate	225	50	1	1
Literate	385	153	0.55 (0.39–0.80)*	0.53 (0.35–0.78)*
Type of the household				
Model household	169	66	0.79 (0.56–1.12)	1.06 (0.71–1.58)
Non-model household	441	137	1	1
Educational status of father				
Illiterate	53	19	1	1
Literate	546	176	1.11 (0.64–1.92)	1.27 (0.81–2.00)
Wealth index				
Poor	191	561	1	1
Medium	375	377	1.15 (0.78–1.71)	1.15 (0.76–1.73)
Rich	188	564	1.94 (1.18–3.20)*	1.80 (1.07–3.03)*

*HDA* Health development army, *HEW* Health extension worker, *VAS* Vitamin A supplementation *Statistically significant at 95% confidence level

## Discussion

The finding of this study indicated that, the cumulative coverage of VAS (75.0%) was approaching the WHO’s minimum acceptable level of 80%. Knowledge of mothers on the importance of VA supplementation, getting information about VAS from frontline health workers and better household economic status were found to be independent predictors of VAS uptake.

In Ethiopia, VAS has been implemented through campaign-based EOS/CHD approaches for a while. Nevertheless, starting from 2011 due to sustainability concerns, the program was integrated into the routine HEP [13]. However, there had been concerns that the transition of the VAS program from campaign-based to routine service delivery may result in decline in VAS coverage [13, 17]. According to the World Bank data, VAS coverage in Ethiopia remained above 80% between 2006 and 2011. But after 2012 – which coincides with shift from campaign-based approach – the coverage dropped below 80% [18]. EDHS 2016 also reported only 45% of children received the supplement in the preceding 6 months of the survey [5]. The finding suggested that the district health office need to closely monitor the VAS distribution in order to maintain the high coverage that used to be attained via campaign-based approach.

We found that nearly three-fourth of the local women were aware of the benefit of VAS for their children. This is consistent to the findings of studies from Kenya [19], Libya [20] and Nigeria [21] that mothers have positive knowledge and attitude towards vitamin A supplement. We also found that mothers having information about the importance of VAS have better uptake of the supplement. Hadzi et al. in Ghana also concluded that caregivers with better knowledge were almost two times more likely to accept vitamin A supplementation to their children [22]. The finding implies that supporting VAS program with strong information, education and communication strategies is likely to increase demand and utilization of the supplement.

Another important finding was that women who received information about the important of VAS from frontline health workers (HEWs and HDA members) were more likely to accept the supplement than those who received the information from health professionals. This might be due to the fact that frontline health workers who are living within the community and share similar sociocultural experiences are more trusted by the community than health professionals working in remote health facilities.

The current study found that children born to literate mothers had 47% reduced odds of receiving VAS than those children having illiterate mothers. The finding is against the widely held believe that maternal education is vital for enhancing health seeking behaviors for child health services. Pertaining to VAS, previous studies reported mixed associations [23–26]. Raut et al. in their analysis of multiple DHS from low- and middle- income countries reported that maternal education was significantly associated with the receipt of vitamin A [23]. The same pattern was also witnessed by the recent Ethiopian DHS 2016 [5] and a study conducted in Nigeria [24]. On the other hand, Haile and his colleagues observed no association between maternal education and receipt of VAS in Ethiopia [25] while Semba et al. reported better VAS utilization by illiterate than literate mothers in Bangladesh [26]. The unexpected association can be due to the reason that illiterate mothers who are more likely to stay at home have a better chance to be contacted by frontline health workers for home-to-home vitamin A distribution. Educated mothers may also have better chance of contacting health professionals, rather than frontline health workers who are directly involved in the distribution of VAS. As the study is also a sample-based study, the finding might also be due to error that emanates from chance.

Wealth index of the household is found to be an important predictor of VAS update. In this study, children from the rich households had almost two times increased odds to receive VAS as compared to those from poor households. According to the Ethiopian DHS 2016, percentage given VAS in the past 6 months increased from 41% among children from the poorest households to 58% to those from richest households [5]. Aremu et al. in Nigeria also identified household wealth status as the only household-level characteristic that significantly affect receipt of vitamin A capsule [27]. Better household wealth standing may improve uptake of the supplement through advancing access to health information and mitigating economic barters for seeking health care.

The following limitations need to be considered while interpreting the limitations of the study. As the study followed a cross-sectional design, it is liable to systematic errors including selection bias and reverse causality and the reported associations may not strongly imply causality. Further, VAS status was determined based on the report of the mother; consequently, recall and social desirability bias cannot be entirely excluded. The fact that the survey was limited to rural villages of the district may theoretically limit the generalizability of the findings for the entire district that has both urban and rural kebeles. The study investigated predictors of VAS from caregivers’ perspective and further studies should look into health system related determinants.

## Conclusion

The coverage of VAS in the study area was 75.0% which is below the WHO’s recommendation and national target of 80%. Knowledge of mothers on the importance of VA supplementation, getting information on VAS from frontline health workers and better household economic status were independent predictors of VAS uptake.

## Supplementary information

**Additional file 1.**

## Data Availability

Supplementary data will be available from the corresponding author upon a reasonable request.

## Abbreviations

AOR: Adjusted odds ratio
COR: Crude odds ratio
CHD: Community health days
DHS: Demographic and health survey
EOS: Enhanced outreach strategy
HDA: Health development army members
HEP: Health extension program
HEW: Health extension worker
OR: Odds ratio
PCA: Principal component analysis
VA: Vitamin a
VAD: Vitamin a deficiency
VAS: Vitamin a supplementation
WHO: World health organization
