# Supplementary material for: Uptake of routine vitamin A supplementation for children in Humbo district, southern Ethiopia: community-based cross-sectional study
Source: BMC Public Health. 2020 Oct 2;20:1500. doi: 10.1186/s12889-020-09617-1 (PMC7532605; doi:10.1186/s12889-020-09617-1)
Supplement: Supplementary file 1 — Additional file 1. [file 12889_2020_9617_MOESM1_ESM.docx]

**ROUTINE VITAMIN A SUPPLEMENTATION COVERAGE, PREDICTORS OF UPTAKE AND ASSOCIATION WITH COMMON CHILDHOOD ILLNESSES IN HUMBO WOREDA: SOUTHERN ETHIOPIA**

**PART ONE: SOCIODEMOGRAPHIC INFORMATION**

| **No** | **Questions for Mothers /care givers** | **Response and code** | **Skip pattern** |
| --- | --- | --- | --- |
| 101 | What is your relation to the child? | 1. Mother  2. Father  3.Other primary caregiver (specify) __________ |  |
| 102 | What is your religion? | 1. Orthodox 2. Muslims 3. Protestant 4. Catholic 5. Other (specify) __________ |  |
| 103 | What is your ethnicity? | 1. Wolayita 2. Sidama 3. Kembata 4. Amhara 5. Oromo 6. Other (specify) __________ |  |
| 104 | How old are you?  (Age of mother/caregiver in completed years) | 1. ______________ years 2. Not sure |  |
| 105 | What is your educational status? | 1. Informal education  2. Formal education (____ grade)  3.Higher education (specify) ___________ |  |
| 106 | What is your marital status? | 1. Married/living together 2. Single 3. Divorced/Separated 4. Widowed | If 1,go to 107  if 2,3,4 go to 109 |
| 107 | How old is the father of the child? | ________ years |  |
| 108 | What is the educational status of father? | 1. Informal education  2. Formal education (__grade)  3.Higher education  4. (specify) __________ |  |

| 109 | What is your occupation? | 1.Farmer  2. House wife  3.Government employee  4. NGO employee  5.Small scale trade  6. Other (specify) __________ |  |
| --- | --- | --- | --- |
| 110 | How many people are living in this household? | __________ |  |
| 111 | Number of under five children in the household | __________ |  |
| 112 | Age of the child in completed months | 1. ______________ months 2. Not sure |  |
| 113 | What is the sex of the child? | 1. Male 2. Female |  |
| 114 | What is the birth order of the child? | __________ |  |

**PART TWO: HOUSEHOLD ECONOMIC STATUS**

| **No** | **Questions for Mothers /care givers** | **Response and code** | **Skip pattern** |
| --- | --- | --- | --- |
| **201** | Do you have a private home? | 1. Yes  2. No | If 1,go to 202  if 2 go to 205 |
| **202** | From what material the roof of the house is made of? | 1. No roof 2. Rudimentary roof (thatch, leaf/mud, plastic sheet etc) 3. Finished roof (asbestos, corrugated iron, metal, …..) 4. Others specify ______ |  |
| **203** | From what material the wall of the house is made of? | 1. Rudimentary walls (bamboo, bamboo/wood with mud, Stone with mud 2. Finished walls (cement, stone with cement, bricks ….) 3. Others specify ______ |  |
| **204** | From what material the floor of the house is made of? | 1. Natural floor (earth/ sand, dung) 2. Rudimentary floor (wood planks, bamboo…..) 3. Finished floor (polished wood, Cement, ceramic …) 4. Others specify__________ |  |
| **205** | Does any member of this household own:  (Read all choices and circle the responses.) | 1. Radio 2. television 3. Mobile telephone 4. Fixed telephone 5. Bicycle 6. Refrigerator 7. Table 8. Chair 9. Bed with mattress 10. Electric Mitad 11. Horse /donkey cart |  |
| **206** | Does any member of this household own any agricultural land? | 1. Yes 2. No | If 1,go to 207  if 2 go to 208 |
| **207** | How many (Local units) of agricultural land do you own? | _____ size  _____ local unit |  |
| **208** | Does this household own any livestock, herds, other farm animals, or poultry? | 1. Yes 2. No | If 1,go to 209  if 2, go to 210 |
| **209** | How money of the following animal does this household own?  If yes give answer in number.  1.Milk cows, oxen or bulls  2. Goats?  3. Sheep?  4. Chickens?  5.Horses, donkeys, or mule | Write 00 if none, write 01 if more than one and write 11 if unknown.  1._______ in no  2.________in no  3.________ in no  4.________ in no  5.________in no |  |
| **210** | What is the monthly income of the household?  (Data collector: Explore all the possible sources of income for the household) | 1_____________ ETB  2. not sure |  |

**PART THREE: KNOWLEDGE ABOUT VITAMIN A AND VAS**

| **No** | **Questions for Mothers /care givers about the child** | **Response and code** | **Skip pattern** |
| --- | --- | --- | --- |
| 301 | Have you ever heard about of VA? | 1.yes 2.No | If 1, go to 302. If 2 go to 304 |
| 302 | Do you know the sources of vitamin A | 1.yes 2.No | If 1,go to 303  if 2 go to 304 |
| 303 | If yes, please mention them.  (Multiple answers possible) | 1. Dark green leafy vegetables  2. Fruits and vegetables with orange or yellow inside  3. Milk and milk products  4.Egg  5.Fish  6.Meat  7.Others specify _________ |  |
| 304 | Do you know the effect of VAD? | 1.yes 2.No | If 1,go to 305  if 2 go to 306 |
| 305 | Could you mention the effects of VAD?  (Multiple answer is possible) | 1 Night blindness  2.Permanent loss of sight  3. Poor physical growth  4. Frequent illness  5. Death  6.Others (specify )_______ |  |
| 306 | Have you ever heard about VAS? | 1.Yes 2.No | If 1,go to 307  if 2 go to 401 |
| 307 | Ever seen VAS capsule? | 1.Yes 2.No |  |
| 308 | What was your source of information about VAS?  (multiple answers possible) | 1.Health professionals  2.Health extension workers  3.Mass media (Television/Radio/newspaper)  4.Health development army members  5. Others ________ |  |
| 309 | How frequently should it be given to children? | _________(times) |  |
| 310 | At what age should the child start VAS? | ________________ |  |
| 311 | Do you believe that VAS has any importance for your child? | 1. Yes 2. No | If 1,go to 312  if 2 go to 313 |
| 312 | What is/are the importance of VAS?  ( more than one response is possible | 1. Prevent blindness  2. Prevent disease  3.Prevent death  4. Helps for growth  5.Other (Specify)_____ |  |
| 313 | Do you believe that VAS creates any problem on the child? | 1. Yes 2. No | If 1,go to 314  if 2 go to part -4 |
| 314 | If yes please specify | 1. ___________________ 2. ___________________ |  |

**PART FOUR: HISTORY OF VAS UPTAKE AND PREDICTORS OF NON-RECIPIENT**

| **No** | **Questions for Mothers /care givers about the child** | **Response and code** | | | **Skip pattern** |
| --- | --- | --- | --- | --- | --- |
| 401 | How far is the nearest health institution?  (one-way estimated walking distance) | __________ minutes | | |  |
| 402 | Vaccination status of the child  ( Data collector: observe the vaccination card) | BCG | 1. Yes | 1. No |  |
|  |  | DPT 3, Penta 3 | 1. Yes | 1. No |  |
|  |  | PCV 2 | 1. Yes | 1. No |  |
|  |  | Rota | 1. Yes | 1. No |  |
|  |  | Measles | 1. Yes | 1. No |  |
| 403 | Did the child has received VAS in the past six month? | 1.Yes 2.No | | | If 1,go to 404  if 2 go to 405 |
| 404 | How did the child get VAS? | 1. CHD Campaign  2.Routine immunization  3.Home visit by health workers  4.Growth monitoring follow ups  5.Sick child visit of health institutions  6.Others | | |  |
| 405 | If not received, for Q.3 why your child did not receive VA? | 1.I don’t think it is good to my child  2.I did not hear the VAS day  3.I forgot VAS day  4.Others specify________ | | |  |

**PART FIVE: ILLNESS HISTORY IN THE PREVIOUS 2 WEEKS AND FACTORS ASSOCIATED WITH THE ILLNESS**

| **No** | **Questions for Mothers /care givers** | **Response and code** | **Skip pattern** |
| --- | --- | --- | --- |
| 501 | Type of household | 1.Model  2.Non model  3.I don’t know |  |
| 502 | Distance from health institutions  (in Kms) | 1. 1-5kms  2. 6-10kms  3. >11kms |  |
| 503 | What is the main source of drinking water for members of your household? | 1. Public standpipe  2.Unprotected dug well  3.Unprotected spring  4. Protected spring  5. Protected dug well  6.Household connection  7.Surface water (river/ lake /pond/stream  8. Rain water  9.Other(specify)_____ |  |
| 504 | What kind of toilet facility do members of your household usually use? | 1.Flush to piped sewer system  2.Flush to pit latrine  3.Ventilated improved pit latrine(VIP)  4. Pit latrine without slab  5.Pitlatrine with slab  6.No facility/bush/ field |  |
| 505 | Did your child dewormed? | 1. Yes 2. No |  |
| 506 | For how many times did your child dewormed in the last 6 months? | _________(times) |  |
| 507 | Do you always have hand washing habit? | 1.yes 2.no | If 1,go to 508  if 2 go to 510 |
| 508 | When did you wash your hands? | 1.After visiting latrine  2. After cleaning child  3. Before feeding  4. Before food preparation |  |
| 509 | What do use to wash hands? | 1. Soap  2. Ashes  3. Other |  |
| 510 | Has the child had diarrhea last 2 weeks? | 1. Yes  2. No  3. Don't know |  |
| 511 | Do you use bed net for the baby? | 1. Yes 2.No | If 1,go to 512  if 2 go to 513 |
| 512 | Did the baby slept under bednet yesterday night? | 1.Yes 2.No |  |
| 513 | Has the child had fever last 2 weeks? | 1. Yes 2. No  3. Don’t know |  |
| 514 | Has the child had cough in last 2 weeks? | 1. Yes  2. No  3. Don't know |  |
| 515 | What is your HOUSEHOLD’s primary source of cooking fuel? | 1.Electriccity  2.Liquefied petroleum gas  3.Natural gas  4.Biogas  5.Kerosene  6.Charcoal  7.Wood  8.Straw/shrubs/grass  9.Animal dung  10. Other(Specify)______ |  |
| 516 | Did your child visited health institution in the last six months for any reason? | 1. Yes  2. No  3. Don't know |  |
